# Microalgae for Bioenergy: Key Technology Nodes

**DOI:** 10.1155/2015/597618

**Published:** 2015-04-27

**Authors:** Ywetta Maleterova, Frantisek Kastanek, Milena Rouskova, Martina Matejkova, Petr Kastanek, Olga Solcova

**Affiliations:** ^1^Department of Catalysis and Reaction Engineering, Institute of Chemical Process Fundamentals of the ASCR, v.v.i., Rozvojova 135, 16502 Prague 6, Czech Republic; ^2^Ecofuel Laboratories, s.r.o., Sazavska 721/17, 12000 Prague 2, Czech Republic

## Abstract

Microalgae have increasingly gained research interest as a source of lipids for biodiesel production. The wet way processing of harvested microalgae was suggested and evaluated with respect to the possible environmental impacts and production costs. This study is focused on the three key steps of the suggested process: flocculation, water recycling, and extraction of lipids. Microalgae strains with high content of lipids were chosen for cultivation and subsequent treatment process. Ammonium hydroxide was tested as the flocculation agent and its efficiency was compared with chitosan. Determined optimal flocculation conditions for ammonium hydroxide enable the water recycling for the recurring microalgae growth, which was verified for the use of 30, 50, and 80% recycled water. For extraction of the wet microalgae hexane, hexane/ethanol and comparative chloroform/methanol systems were applied. The efficiency of hexane/ethanol extraction system was found as comparable with chloroform/methanol system and it seems to be promising owing to its low volatility and toxicity and mainly the low cost.

## 1. Introduction

Microalgae have been considered as an alternative renewable energy source for biodiesel which could substitute oil from the seed crops. The production of biodiesel from algae has several advantages: high biomass productivity, high content of oil up to 80%, oils with the high lipid content, the need of nonarable lands for their growth, capability of growth in salt water and waste streams, and capability of solar light and CO_2_ gas utilizing as nutrients. Therefore, a number of scientists have been reported application of microalgae for biodiesel production [1–4]. Biodiesel produced from microalgae belongs to the third generation of biofuels which overcomes disadvantages of the first (biodiesel produced from palm oil, coconut, sunflower, etc.) and the second (biodiesel produced from* Miscanthus*,* Jatropha*, salmon oil, tobacco seed, etc.) generation of biofuels [5]. Mainly, the microalgal production does not compete for land with food crops [6, 7].

Nevertheless, cost of the microalgal biodiesel production is relatively higher compared to other feedstocks owing to the high energy consuming drying process of harvested microalgae [8]. The most common harvesting methods include sedimentation, centrifugation, filtration, chemical flocculation, and of course drying before the oil extraction [9, 10]. Mechanical press, the use of chemical process, and supercritical extraction rank are among the usually applied extraction techniques. All mentioned processes are energy consuming or could have a negative environmental impact.

From those reasons our work is focused on the new way of the oil production from microalgae. The completely wet treatment, which enables to release the energy consuming drying step, has been studied. The individual key technology nodes have been also evaluated with respect to the possible environmental impacts.

## 2. Materials and Methods

### 2.1. Laboratory Cultivation

The applied strain of microalgae,* Chlorella vulgaris* Beijerinck 256, was obtained from the Culture Collection of Autotrophic Organisms (CCALA) of Institute of Botany, ASCR, v.v.i., and contained about 37% of oils. The microalgae were cultivated in the growth medium that includes 600 mg/L urea, 1480 mg/L KCl, 988 mg/L MgSO_4_·7H_2_O, 340 mg/L KH_2_PO_4_, 11 mg/L CaCl_2_·6H_2_O, 18 mg/L EDTA Fe/Na, 3.09 mg/L H_3_BO_4_, 1.18 mg/L MnSO_4_·4H_2_O, 1.4 mg/L CoSO_4_·7H_2_O, 1.43 mg/L ZnSO_4_·7H_2_O, 1.4 mg/L CuSO_4_·5H_2_O, and 0.88 mg/L (NH_4_)_6_Mo_7_O_24_·4H_2_O according to the literature [11] with the modification to use urea and KCl instead of KNO_3_. The laboratory cultivation units consisted of the glass cylinders (inner diameter 36 mm, height 500 mm), which were placed in a thermostatic bath (29°C) with the continuous illumination by the panel dimmable fluorescent lamps (Philips Master TL-D 36W/840, type warm white) [12] allowing the adjustment of the incident light intensity from 16 to 780 *μ*mol/(m^2^s). The cylinders were “aerated” by a mixture of air and CO_2_ (2% v/v). Volume of the algal suspension in each cylinder was 300 mL and each cylinder was supplied by gas at a flow rate of 15 L/h. Microalgae were cultivated for 11 days to characterize their growth rates. The microalgal growth in recycling water was tested under continuous illumination with addition of 30, 50, or 80% of recycling water in two (for 80%) or three (for 30 and 50%) consequent experiments.

### 2.2. Laboratory Flocculation

Chitosan and ammonium hydroxide have been used as flocculation agent. In all experiments the initial concentration of algae varied between 2.70 and 3.25 g/L of dry weight. The algae suspension was placed to graduated cylinders in the amount of 250 mL. To these samples 0.2 to 19 mL of ammonium hydroxide solution (26%) was added to obtain solutions with pH between 8.5 and 10.5. Chitosan diluted in the distilled water was dosed in the amounts 5, 10, 15, and 20 mg/L to obtained pH of value 7. Suspension was intensively agitated for 10 s and in the time intervals 1, 5, 10, 15, and 20 minutes was sampled in the distance of 20 mm below the water level in the graduated cylinder. Samples were analyzed on the photospectrometer SPEKOL 1300 and the optical density (OD) was measured and converted by the calibration curve to the concentration.

### 2.3. Extraction Experiments

Dry matter in algal suspension was determined before each extraction to assure the exact content of the dry algal biomass in solution (105°C, 7 h to the constant weight). In each experiment, microalgal suspension (containing 10 g dry biomass) was treated by the single-stage extraction with the hexane/ethanol mixture (2/3 v/v) at 1/15 (dw/v) ratio in continuously stirred Erlenmeyer flask for 4 h under inert atmosphere in the absence of light at the ambient temperature. Individual phases (liquid organic extract, water phase, and solid biomass) were separated from the obtained solutions by filtration process on the nutsch. The upper organic phase (extract) was sucked off. The solvent was then removed from the organic phase by rotary evaporation at 40°C after which the total lipid content (extractable part) was determined gravimetrically. An aliquot of the dry extract was taken for the following analysis.

Analysis of fatty acid (FA) profile in the extracts of the tested microalgae was performed at the Department of Food Analysis and Nutrition of the Institute of Chemical Technology Prague. Accredited (ISO 17025) gas chromatographic (GC) method was used. Briefly, following the release of FAs from ester bonds by saponification, their methylation was performed. Target analytes were separated on capillary column and detected by the flame ionization detector (FID). Quantitative determination was carried out by the inner standard technique performed by direct comparison of the addition of the inner standard nonadecane acid (C19:0).

## 3. Results and Discussion

**Figure 1:**
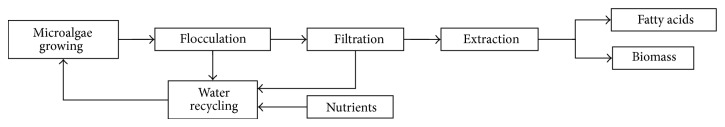
Scheme of the algal treatment with the individual steps.

The whole process of the oil extraction is schematically illustrated in Figure 1, where the schema with individual steps is depicted. Flocculation, water recycling, and extraction belong to the most energy consuming steps and/or could have a negative impact on the environment [10].

### 3.1. Flocculation

The basic task in the first step of algae suspension treatment past its cultivation is elimination of the major part of water from the algal suspension. Microalgae before harvesting include the really high amount of water. Usually the concentration of algae achieved maximally about 30 g/L of dry weight in dependence of the used growing technology [13]. Therefore, flocculation of algae by coagulants with subsequent separation has been applied to obtain suspension with high concentration of algae.

As a flocculation agent chitosan has been usually used. Nevertheless, to decrease the cost of this operation and enable water recycling the other flocculation agents have been searched and tested [14]. The ammonium hydroxide seems to be the promising coagulant. Application of ammonium hydroxide as the flocculation agent causes the increase of the solution pH value which allows the magnesium hydroxide and/or calcium hydroxide generation.

Hydroxides cover the surface of algae and in combination with neutralization and its adhesion on the cell surface cause formation of heavier flocks which can easily sediment. The ammonium hydroxide seems to be the promising coagulant. Application of ammonium hydroxide as the flocculation agent causes the increase of the solution pH value which allows the magnesium hydroxide and/or calcium hydroxide generation.

**Figure 2:**
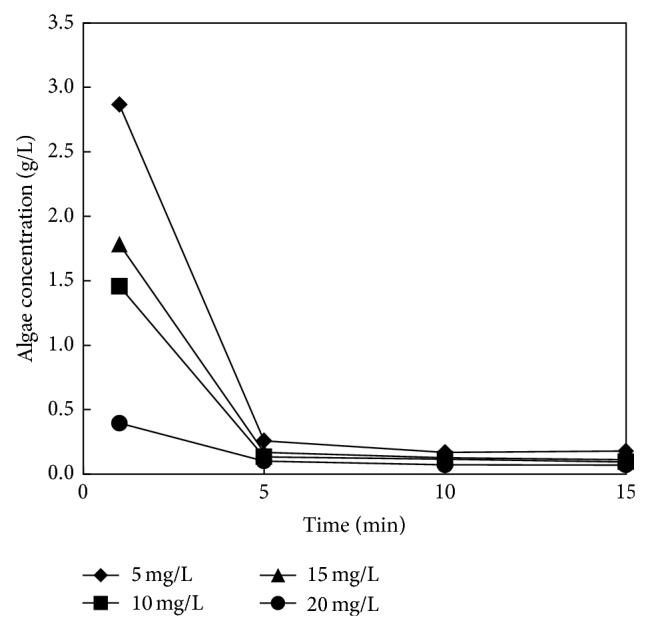
Flocculation by chitosan.

**Figure 3:**
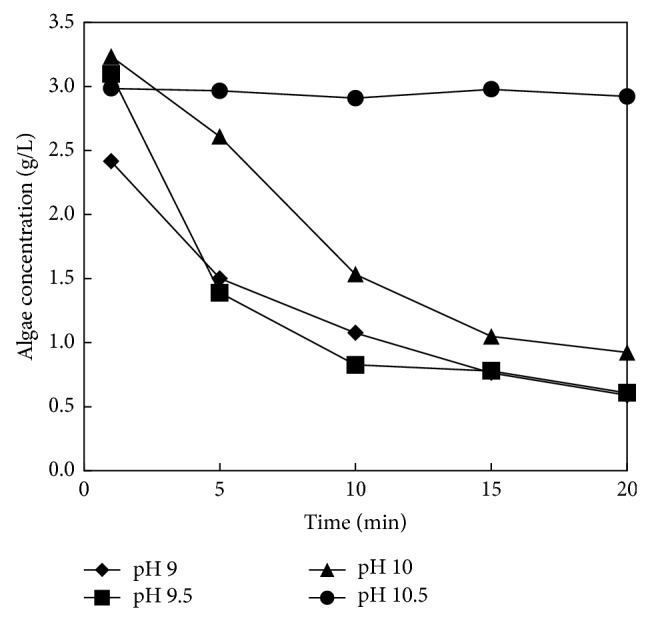
Ammonium hydroxide flocculation.

**Figure 4:**
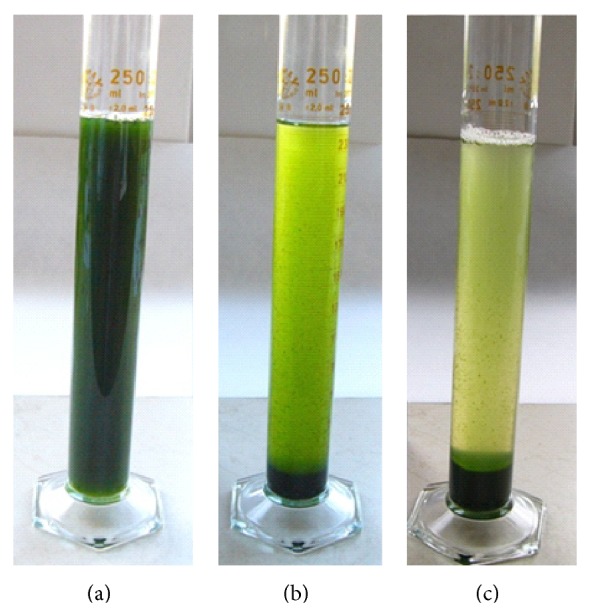
(a) Without any flocculation agent, 0 min; (b) with NH_4_OH, pH 9, after 20 min; (c) with chitosan 5 g/L, after 20 min.

Rate of flocculation for chitosan and ammonium hydroxide as the flocculation agent is shown in Figures 2 and 3. It can be easily seen that flocculation rate for chitosan depends only slightly on chitosan concentration; see Figure 2. During first 5 minutes the flocculation efficiency of chitosan achieved minimally 95% for the whole range of the tested concentrations (5–20 mg/L). On the contrary, flocculation by ammonium hydroxide significantly depends on pH value of solution and thus on the ammonium hydroxide concentration; see Figure 3. The efficiency about 86% was achieved for pH 9 during 20 minutes. Moreover, the increasing pH value decelerates the flocculation rate and at pH 10.5 the flocculation is completely stopped. For better illustration photos after 20 minutes of flocculation for various agents are shown: Figure 4(a), without flocculation agent; Figure 4(b), ammonium hydroxide at pH 9; and Figure 4(c), chitosan with concentration 5 g/L. Water from both flocculation experiments was decanted and thickened algal solutions were filtrated on the nutsch.

The average efficiency of ammonium hydroxide as the flocculation agent is about 80%; however, it provides the price 3–6 USD per ton of dried biomass while efficiency of chitosan as flocculation agent is higher (95%), but the price per ton of dried biomass is 30−60 USD. Nevertheless, the highest price was estimated for the direct centrifugation of harvesting algae without flocculation step at 60–300 USD per ton of dried biomass.

### 3.2. Water Recycling

It must be emphasized that ammonium hydroxide not only is the low cost and effective flocculation agent but also brings into the algal water solution only the biogenic elements. This fact is significant for the next step of algal treatment process, the water recycling.

**Figure 5:**
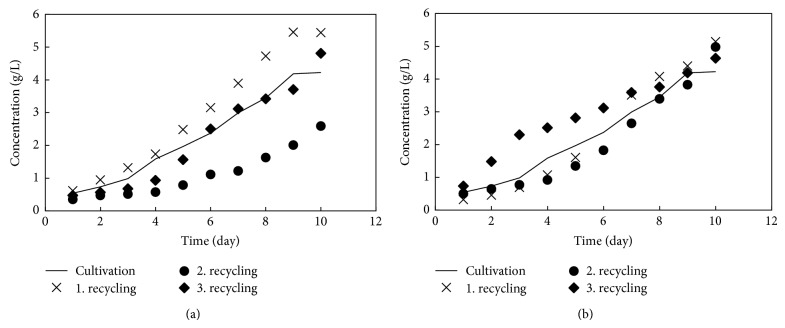
Cultivation growth curve with recycling water: 50 and 80%.

The influence of recycling water was tested on the cultivation growth curve of* Chlorella vulgaris 256* with 50 and 80% of recycled water. In Figure 5 the original cultivation growth curves (line) are compared with the repeated growth curves in water with addition of 50 and 80% of recycled water. It can be seen that utilization of the recycled water has no influence on the growth curves of algae. It is the important fact to the process economy and also to the environmental impact, since during the microalgae treatment the huge amount of water is produced.

### 3.3. Extraction

One of the crucial key process nodes of the energy production from harvesting algae is the extraction step. Usually, extraction of dry algal biomass of algae has been performed [15–17]; however this work is focused on wet way extraction. It can bring the significantly lower energy cost and no degradation of valuable fatty acids during the extraction process. Data about the wet extraction process appear only rarely in the literature. Halim et al. [18] obtained the comparable lipid yields by hexane extraction from either dried microalgal powder or wet microalgal paste.

Hexane is often recommended for extraction of the lipid fraction from microalgae. Its advantage is the chemical stability, almost nonsolubility in water, and relatively low boiling point, which is favourable for separation/regeneration.

Generally, hydrocarbons require the use of the other solvents as deemulsifiers to prevent formation of foams and stable emulsions, that is, reduction of interfacial tension. Therefore, ethanol was added to the extraction system due to its relatively low boiling points and favourable regeneration from water phase. For determination of the total lipid content the solvent system of chloroform/methanol mixture has been applied by Bligh and Dyer [19]. This method was primarily developed for determination of lipids in the cod muscle. Therefore, it possesses some limitation for samples of plant/algae origin which contains pigments and the other soluble substances. This solvent system significantly increases the proportion of extractables and its toxicity is not environmental friendly as well. Nevertheless, Bligh and Dyer [19] method has been usually applied as a comparative procedure.

**Figure 6:**
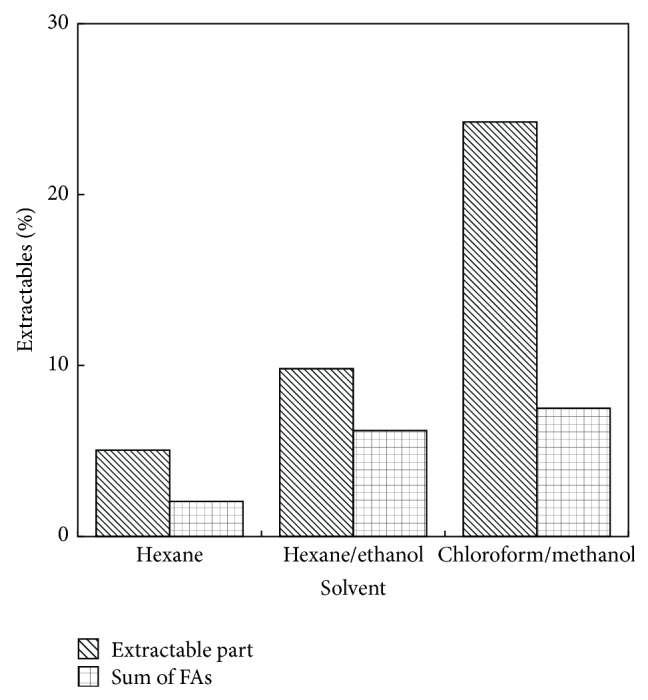
The effect of solvent mixture composition on lipid (extractable part) and sum of FAs content related to dry matter.

**Figure 7:**
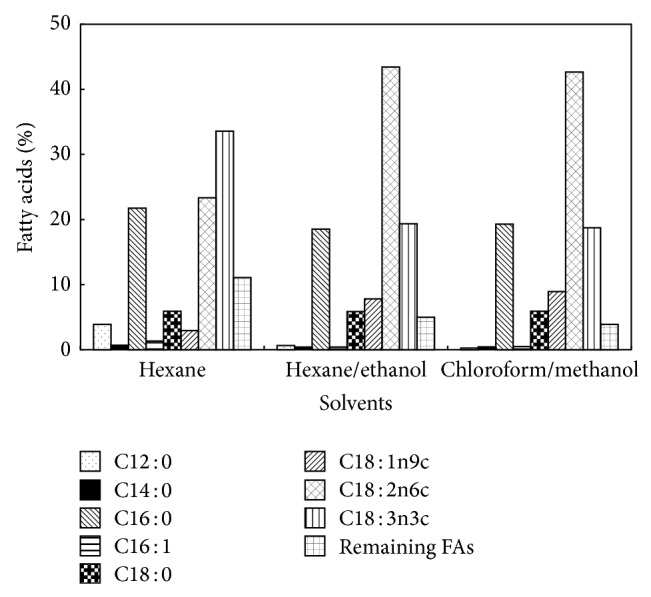
The effect of solvent mixture composition on the profile of fatty acids.

First, the effect of solvent polarity on the extractable part and the total fatty acids in fresh microalgae was tested in this study (Figure 6). Maximal extractable part was obtained by chloroform/methanol system (24.3%) which contains except lipids the other soluble components, for example, pigments. It can be clearly seen that hexane/ethanol system provides the same amount of the extraction fraction of the total lipids (FAs) as system chloroform/methanol.  Figure 7 shows that also profiles of FAs are comparable for both systems. Thus, hexane/ethanol mixture, which is the environmental friendly solvent system, was proved to be a suitable alternative for extraction of lipids/FAs. This system possesses relatively high extraction capacity, low volatility and toxicity for humans as well as environment, and moreover the low cost. The highest proportional content of fatty acids in the lipid fraction was included by hexane/ethanol mixture (63.2%).

Pure hexane showed the lowest extraction capacity for total extractables and extracted 40.6% FAs in the lipid fraction.

These experiments confirmed the conclusion of Halim et al. [18] that efficiency of the microalgal extraction by the wet way is comparable with the dry way and with addition of ethanol it provides the same lipid yields. Moreover, profiles of FA are not affected by presence of water in extracted biomass. It influences only the amount of extraction solvent related to dry microalgae. Hexane/ethanol extraction system enables the subsequent utilization of the residual biomass, for example, as the poultry feed supplement.

## 4. Conclusions

The three key steps, flocculation, water recycling, and extraction of microalgal treatment for lipid production, have been suggested and evaluated with respect to the possible environmental impacts and production costs. To avoid the energy consuming drying step the completely wet way treatment has been applied. It was verified that ammonium hydroxide can serve as the efficient and the low cost flocculation agent. The optimal flocculation conditions were determined at pH 9. Moreover the application of ammonium hydroxide brings into the algal water solution only the biogenic elements and thus enables the water recycling for the recurring microalgae growth. Water recycling was verified for the use of 50 and 80% recycled water.

It was confirmed that extraction of the wet microalgae can be applied instead of the dry microalgal extraction, which enables to release the energy consuming drying step. The efficiency of hexane/ethanol extraction system was found as comparable with chloroform/methanol system: the comparative method. Moreover, not only the amount of the extraction fraction of the total lipids but also the profiles of fatty acids were the same. Except of the relatively high extraction capacity, hexane/ethanol extraction system possesses the low volatility and toxicity for humans as well as environment and mainly the low cost.

The wet way processing of the harvested microalgae for biodiesel production seems to be the low cost promising biotechnological application with the minimal environmental impact.
